# Direct Comparison of Herbicidal or Biological Treatment on *Myriophyllum spicatum* Control and Biochemistry

**DOI:** 10.3389/fpls.2018.01814

**Published:** 2018-12-10

**Authors:** Michelle D. Marko, Jason C. White

**Affiliations:** ^1^Department of Biology, Concordia College, Moorhead, MN, United States; ^2^Department of Analytical Chemistry, The Connecticut Agricultural Experiment Station, New Haven, CT, United States

**Keywords:** carbon allocation, Eurasian watermilfoil, *Euhrychiopsis lecontei*, 2,4-D, fluridone

## Abstract

*Myriophyllum spicatum* or Eurasian watermilfoil (hereafter, milfoil) is among the most problematic invasive aquatic plant species throughout much of North America. *M. spicatum* infestations can result in reduced diversity and abundance of native plant populations. Control of the invader is essential to promoting healthy ecosystems. Several treatment alternatives are available for milfoil control, although cost and efficacy vary significantly, with some treatments resulting in more harm to the native population than no treatment at all. A series of field-based microcosms containing actively growing milfoil were constructed in order to directly compare the impact of two herbicides (2,4-dichlorophenoxyacetic acid and fluridone) and the milfoil weevil (*Euhrychiopsis lecontei*) on weed control and plant biochemistry. Herbicide concentrations in water, plants, and sediments were monitored, as were weevil population dynamics and resulting invertebrate damage to milfoil stems. The impact of the different treatments on levels of polyphenols, carbohydrates, ash, and overall carbon and nitrogen levels in the milfoil were determined. Total biomass of the untreated milfoil increased by more than 2.7-fold during the 53-day experimental period. Conversely, the biomass of milfoil subjected to chemical or biological treatment either remained constant or decreased significantly during the experiment. The herbicide 2,4-D resulted in nearly 100% milfoil mortality by day 20, whereas fluridone toxicity was significantly slower but reached 75% by the end of the trial. Similarly, milfoil growth in the weevil-amended tanks was somewhat erratic but by the end of the trial, the total plant biomass was 71% less than that of un-amended controls. Although the total biomass remaining at the end of the fluridone and weevil treatments was similar, the carbohydrate and starch content of the shoots in the insect treatment were nearly 4.6- and 4.8-fold greater, respectively, than that of the herbicide treated plants. The higher starch content in insect-treated plants could lead to increased autofragmentation and spread of *M. spicatum*. However, herbicide treatments are frequently required for several years. Therefore, integrated pest management, which combines the long-term benefits of biological controls with the short-term benefits of herbicides may provide the best solution to the control of *M. spicatum* and the conservation of native plants.

## Introduction

Eurasian watermilfoil [*Myriophyllum spicatum* L. (Haloragaceae); hereafter, milfoil] is among the most widespread and aggressive invasive aquatic plants in North America (Smith and Barko, 1990). The native range of *M. spicatum* is throughout Asia, Europe, and parts of Africa; although its current distribution includes 48 states of the United States, as well as large sections of Canada (EDDMapS, 2015). This broad range is actually quite remarkable; the plant seems to grow just as well after over-wintering under the ice in New England as it does in the year long-growing season of the south. Milfoil is a submersed, rooted perennial dicotyledon that is typically found at average water depths of 0.5–3.5 m. Once introduced to a water body, *M. spicatum* often grows to form dense stands that outcompete native plants (Smith and Barko, 1990; Madsen et al., 1991). *M. spicatum* beds can also impede navigation of waterways, and makes boating and swimming dangerous. Direct negative impacts on bird and fish populations have also been documented (Aiken, 1979; Madsen et al., 1995; Schultz and Dibble, 2012; Simbanegavi et al., 2018). *M. spicatum* reproduction is primarily vegetative, largely explaining its potential for explosive population growth. Spread among lakes can occur by a number of means, including loose vegetation attached to boats/trailers and fragments carried by avian species further threatening native plant populations (Madsen et al., 1988).

A variety of treatment options are available to manage *M. spicatum*, although the methods can vary significantly in terms of both efficacy and cost. Identifying cost-effective and ecologically sensitive methods of control can reduce impacts on native plant populations. Herbicides are perhaps the most widely employed and effective technique in controlling unwanted aquatic vegetation in the United States (Gettys et al., 2014). There are several contact herbicides listed for aquatic use and although these products cause rapid plant die back, re-growth from unharmed root tissues can be significant. As such, systemic herbicides that impact all plant tissues often provide better long-term control. Two common systemic aquatic herbicides used in the United States are 2,4-dichlorophenoxyacetic acid (2,4-D) and fluridone (Bugbee et al., 2003; Bugbee and White, 2005; Madsen et al., 2014). Whole lake treatments are frequently recommended due to concerns over the chemical dissipation prior to control and as a result, costs can be significant for larger water-bodies. Under some conditions, carefully executed spot treatments can be effective, thereby minimizing impacts to native communities, but permitting and public acceptance can still be issues of concern with herbicides. Herbicides are generally considered to be the most efficient and effective means of *M. spicatum* control in the United States (Madsen et al., 2014). However, use of herbicides to control submersed aquatic plants is illegal in Europe and herbicide resistance is present in some hybrid milfoils (Poovey and Getsinger, 2007). Herbicides can also result in an abundance of decaying plant biomass in a lake. Therefore understanding the direct impacts of herbicides vs. potential biological control organisms is important to determining the best control options for each unique situation.

There is also much interest in biological control options for invasive aquatic plants such as *M. spicatum* (Reeves et al., 2008; Havel et al., 2017). Three insects have been associated with declines in *M. spicatum* populations (see review by Newman, 2004). *Cricotopus myriophylli* Oliver (Diptera: Chironomidae) has been shown to consume *M. spicatum* meristems and suppress growth. *Acentria ephemerella* (Denis and Schiffermüller) (Lepidoptera: Pyralidae) has been shown to decrease *M. spicatum* biomass in mesocosm experiments and is associated with dramatic decreases in *M. spicatum* biomass in New York lakes (Johnson et al., 2000; Gross et al., 2001). *Euhrychiopsis lecontei* (Dietz) (Coleoptera: Curculionidae) may control *M. spicatum* populations and is particularly interesting as recent studies have shown that the weevil actually prefers the invasive *M. spicatum* to its native host native milfoil (*M. sibiricum* Komarov) (Haloragaceae) (Solarz and Newman, 1996, 2001; Sheldon and Jones, 2001). However, due to the lag-time for weevil response to increases in the *M. spicatum* population, native diversity can be harmed and milfoil populations can become problematic.

The goal of this current study was to directly compare the impact of three different treatment options on *M. spicatum* under controlled conditions. Two frequently applied systemic herbicides listed for *M. spicatum* control, 2,4-dichlorophenoxyacetic acid and fluridone, were compared to an introduced biocontrol agent, *E. lecontei*. The impact of these treatments on milfoil biomass, as well as tissue-specific content of polyphenols, carbohydrates, starch, ash, and carbon:nitrogen ratios was determined. A direct comparison of the impacts of the milfoil weevil vs. herbicides in a controlled setting has not been previously performed. This comparison is important piece of determining the optimal means of control in order to reduce the use of herbicides, and encourage long-term planning and restoration.

## Materials and Methods

### Microcosm Design

Experimental microcosm tanks were established at Lockwood Farm in Hamden, CT, United States. Sixteen 387 L (132 cm × 69 cm × 71 cm, L × W × H) tanks (Rubbermaid) were arranged in a randomized block design. Each tank was amended with 32 plastic pots (10.5 cm × 10.5 cm × 11 cm) containing a lake sediment/agricultural loam (50:50) mix. De-chlorinated tap water was slowly added to a depth of 30 cm. *M. spicatum*, collected from Lake Quonnipaug, Guilford, CT, United States (Lat. 41.388923°, Lon. −72.698632°), was inspected for damage, cleaned of invertebrates, cut to 20 cm lengths and planted at a density of four stems per pot. The plants were left to root and establish for approximately 3 weeks. Floating or unestablished stems were replaced daily. Algal growth was minimal throughout the course of the experiment, but removed from tanks by hand when necessary. Water levels were maintained in tanks at approximately 60 cm or approximately 350 L.

### Experimental Design

The impact of chemical and biological treatments on plant growth was determined using three treatments with an un-treated control. The commercially available formulation of 2,4-dichlorophenoxyacetic acid (2,4-D) recommended for *M. spicatum* control is Navigate^®^ (Applied Biochemists, Milwaukee, WI, United States); the material is a pelletized butoxyethylester formulation with 27.6% active ingredient. A second herbicide recommended for *M. spicatum* control is fluridone, which is commercially available in liquid form as Sonar A.S.^TM^ (SePRO, Carmel, IN, United States). Both herbicides were added to the experimental tanks at the application rates listed on the label for each product. For Navigate^®^, an application rate of five pounds per 2000 ft^2^ or 1.8 g per tank is listed to control *M. spicatum* within 2–3 weeks. With 350 L of water; this was equivalent to an initial 2,4-D concentration of 1.45 mg/L. For fluridone, a concentration of 40 μg/L in water for 1–2 months is required for control; as such, 36 μL was added to each tank. Twenty eight days after this initial application, the fluridone concentration had declined to 12 μg/L. At this time, an additional 18 μL of fluridone was added to each tank to “bump up” the concentration; this technique is recommended by the manufacturer to sustain effective herbicide levels for the required control period. Aqueous herbicide concentrations were monitored over the duration of the experiment (see below). Each treatment had four replicate tanks.

Cultures of the milfoil weevil *E. lecontei*, collected from Dooley Pond, Middletown, CT, United States (Lat. 41.5116712°, Lon. −72.6679638°), were established in our laboratory approximately 1 month prior to treatment. In order to achieve the desired density of 1–2 weevils per stem known to control *M. spicatum* (Newman, 2004), ten adult weevils (at least five females) were added to each tank and allowed to oviposit for 10–14 days. *M. spicatum* meristems were inspected daily to achieve the final density of 1–2 eggs per stem. Weevils typically emerge from pupation after about 25 days (Mazzei et al., 1999); populations were monitored over the subsequent 40 days.

Despite careful inspection and removal of invertebrates at the start of the experiment, some invertebrates remained on the plants or in the collected sediment. Therefore, the damage from weevils along with characteristic damage from other invertebrates, such as snails, caddisflies, *A. ephemerella*, and *Paraponyx* sp. was noted. Invertebrate presence and damage was determined through individual inspections of all stems collected from all treatments biweekly. Weevil presence was enumerated by life stage (egg, larva, pupa, adult). Total weevil impact was assessed by presence of any life stage or characteristic larval damage.

### Plant Growth

On a monthly basis, at least two pots of four stems were randomly selected from each replicate tank for determination of biological and chemical parameters. Average values per tank were used for analyses. Biological parameters monitored included weevil density, longest stem length (cm), longest root length (cm), wet and dry mass (mg), as well as a determination of the number of meristems per plant. Plants were separated into plant parts: the top 15 cm (“tips”), the remaining above ground portion (“middle”), and roots. In biomass analyses, the above ground portions (tips and middle) were combined as above ground treatments.

### Plant Chemical Analysis

Plants were separated into parts (tips, middle, and roots), lyophilized, ground to a fine powder with a coffee grinder (Braun) or mortar and pestle, and weighed for chemical analyses. Chemical analyses included determination of the percent composition of carbon, nitrogen and the elemental carbon:nitrogen ratio, carbohydrates, starch, organics, polyphenols, and ash. Milfoil stems were analyzed for carbon and nitrogen content with a PerkinElmer Series II, CHN/O Analyzer 2400 (Norwalk, CT, United States). Molar ratios of C:N were calculated and used in statistical analyses. Total phenolic compounds (TPC) were determined with the Folin-Ciocalteau assay using tannic acid as the standard (Bowyer et al., 1983). Results were expressed as tannic acid equivalents based on dry mass (TAE). Carbohydrate and starch content were determined by tissue digestion followed by HPLC analysis according to the method of Gent (1984); however, for carbohydrates, the flow rate was modified to 0.6 ml/min. Glucose, fructose, and sucrose concentrations were measured separately and summed for carbohydrate concentrations. Ash content was determined by determining the mass of a dry plant sample before and after heating at 350°C for 24 h.

### Herbicide Analysis

The concentrations of 2,4-D and fluridone in the water, sediments, and plant tissues were monitored over the 53 days of the trial. For water, 250 ml samples were taken from the 2,4-D treated tanks on 1, 2, 6, 8, 14, 21, 35, and 53 days after initial herbicide application. To cleave the ester group from the molecule, NaOH (6 M) was added to the sample in order to raise the pH of the water to 12. After 1 h, the pH was lowered to 2 by the addition of 6 M HCl. For fluridone, 250 ml water samples were taken 1, 2, 6, 8, 14, 21, 28, 35, 53, 68, and 84 days after application. Two hundred milliliters aliquots of water containing either 2,4-D (acid) or fluridone were subjected to solid phase extraction (SPE) using Bakerbond 6-ml cartridges packed with 500 mg octadecylsilane (C_18_) on vacuum manifold. After sample elution, air was drawn over the cartridges for 5 min. The cartridges were then centrifuged at 500 rpm for 10 min to remove any remaining water. Each cartridge was eluted with 2.0 ml of methanol that was collected in a chromatography vial. To determine herbicide levels in the sediment, approximately 3.0-g of air dried sediment was weighed in 20 ml amber vials with Teflon-lined caps. The vials were amended with 15 ml of hexanes and were heated at 65°C for 4 h. A 1-ml aliquot of the supernatant was passed through a glass microfiber filter (0.2 μm, Laboratory Science Inc., Sparks, NV, United States) and collected in a chromatography vial. To determine the herbicide content of sampled *M. spicatum*, 1–5 g of vegetation (root or shoot) was added to a 40 ml amber vial with a Teflon-lined cap. The vials were amended with 20 ml of hexanes and were heated at 50°C for 2 h. One-ml aliquots were filtered and collected as described above. All hexanes extracts of water, sediment, and vegetation were stored at −4°C prior to analysis.

### Herbicide Quantitation

In water, 2,4-D was quantified by high pressure liquid chromatography (HPLC) (Hewlett-Packard 1100 series) using a Supelco Discovery column (5 μm C_18_, 4.6 mm × 250 mm) with a mobile phase of 3:2 methanol/1% glacial acetic acid. The mobile phase flow rate was 1 ml/min and detection was by ultraviolet diode array at 280 nm. In sediments and plants, the quantitation of 2,4-D was determined on an Agilent (Avondale, PA, United States) 6890 gas chromatograph (GC) with a ^63^Ni micro-electron capture detector (ECD). An SPB-1 film (Supelco, Bellefonte, PA, United States) GC column (30 m × 0.53 mm × 0.5 μm) was used and the oven program was as follows: 75°C initial temperature ramped at 5°C/min to 206°C, then ramped at 20°C/min to 280°C. The injection port was maintained at 250°C and a 2-μl splitless injection was used. The carrier gas over the column was He. The ECD was maintained at 325°C and the make-up gas to the detector was 5% CH_4_ in Ar at 60 ml/min. Fluridone was quantified using an Agilent (Avondale, PA, United States) 6890 gas chromatograph (GC) with an Agilent 5973 mass selective detector (MSD) in selective ion monitoring (SIM) mode; the target ion was 328. The column (30 m × 250 μm × 0.25 μm) contained a MDN-12 film (Supelco, Bellefonte, PA, United States) and the GC program was 180°C initial temperature, held for 1 min, then ramped at 10°C/min to 280°C and held for 14 min. A 2-μl splitless injection was used, the injection port was maintained at 300°C and the MS detector was maintained at 280°C.

### Statistical Analysis

Data were analyzed with SAS 9.1 (SAS Institute Inc., Cary, NC, United States). Milfoil biomass data were tested for normal distribution (K–S test or Shapiro–Wilk test) and variance homogeneity (Levene’s test). Extreme outliers were removed. A log transformation was performed with biomass data that did not meet the tests for normality and homogeneity. Response variables were analyzed separately by plant part. Statistical differences were determined by two-way ANOVA (GLM procedure) using treatment, collection date (DAT, days after treatment) and treatment by DAT interaction. When date was significantly different, response variables were analyzed separately by treatment for each DAT. The 2,4-D treated plants were dead by the last collection of plants and were excluded from analyses of samples on the final collection. The presence of snails, and caddisflies was assessed using a log-linear model (GENMOD procedure, Poison distribution) with treatment and collection date as explanatory variables.

## Results

### Treatment Efficacy

**FIGURE 1:**
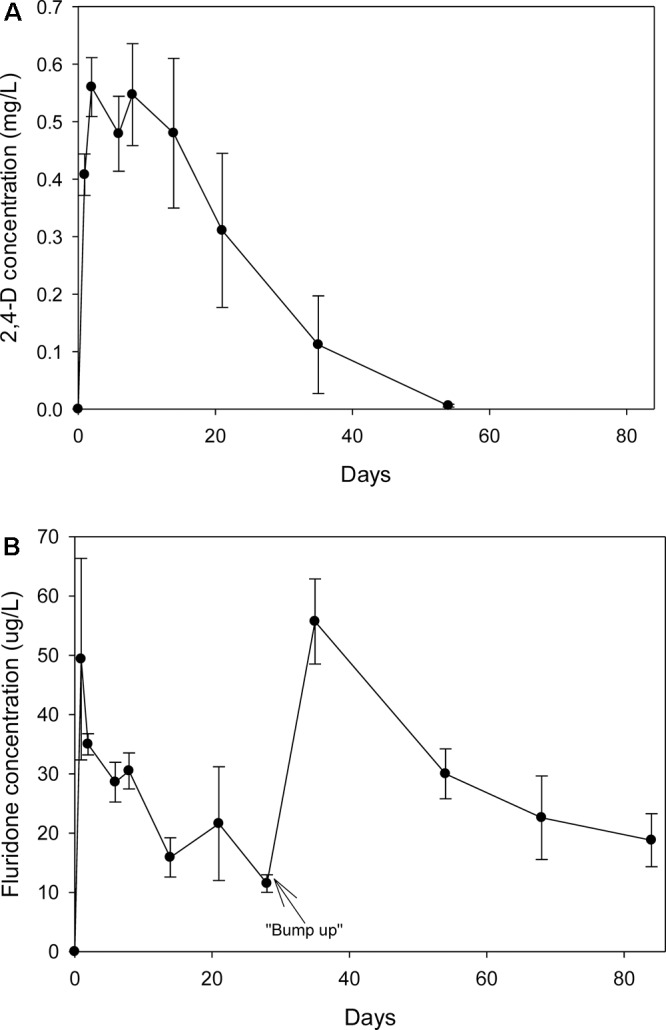
**(A)** Concentration of 2,4-D (Navigate^®^) in the water from tanks treated with the herbicide on 7/24/2007. **(B)** Concentration of fluridone (Sonar^TM^) in the water from tanks treated with the herbicide on 7/24/2007. Fluridone residuals were measured past the regular experiment to ensure that the dose was maintained for at least 60 d.

The recoveries of spiked 2,4-D and fluridone from water via SPE were 103 ± 5.16% and 135 ± 13.6%, respectively. Both un-spiked control water samples and pre-treatment tank water samples had non-detectable levels of the herbicides. One day after application, the 2,4-D levels were 0.41 mg/L; at day eight, the concentration was 0.55 mg/L and subsequently began to decline (Figure 1A). By day 53, the levels were only slightly above the detection limits. One day after application, the fluridone concentration reached 49 μg/L but steadily declined to 12 μg/L on day 28 at which point additional fluridone was added (Figure 1B). By day 38, the concentration had risen to 56 μg/L and steadily decreased to 19 μg/L by day 84. There were non-detectable levels of both herbicides in the water from control and weevil tanks.

Neither herbicide was detected in sediment samples taken during the exposure period. In addition, 2,4-D was not detected in any of the harvested vegetation. Fluridone was detected in vegetation samples harvested 14 and 48 days after treatment. The average fluridone concentration (dry weight) in harvested milfoil shoots and roots was 31.8 (± 20.3, ± 1 standard deviation) ng/g (dry weight) and 14.3 (± 20.3, ± SD) ng/g (dry weight), respectively. There were no significant differences in the root or shoot fluridone content between the two sampling periods.

Stable populations of *E. lecontei* formed in all inoculated tanks reaching the target density of 2 weevils/stem (2.08 ± 0.376, mean ± 1 SE). Snails were found in all tanks in low numbers throughout the experiment (0.49 ± 0.12) and were not significantly different by treatment or date after treatment (χ^2^ = 121.5, df = 131, *P* > 0.1). Caddisflies were found in many tanks and removed during daily inspections (0.12 ± 0.012) and were not significantly different by treatment or date after treatment (χ^2^ = 56.4, df = 131, *P* > 0.1).

### Plant Growth

**FIGURE 2:**
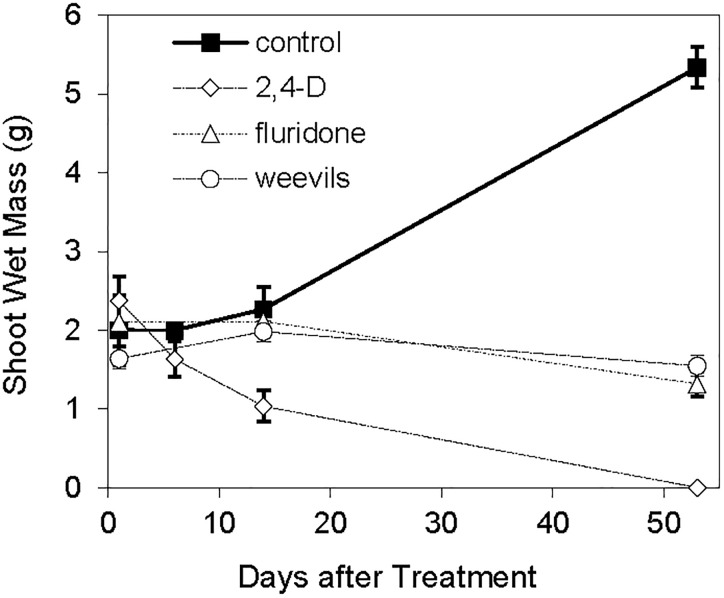
Wet mass of *M. spicatum* above ground plant tissues.

The log-transformed plant biomass (wet weight) were significantly higher for control treatments than for the herbicide or weevil treated tanks (Table 1 and Figure 2). During the course of the 53 day experiment, the above-ground wet biomass of the control plants increased by 2.7-fold. Conversely, the wet mass of shoots exposed to weevils or fluridone decreased by 5.5 and 37%, respectively. Plants exposed to 2,4-D lost 57% of their mass within the first 2 weeks and were completely dead by the end of the trial. Similarly, the wet root biomass was highest for control plants, intermediate for fluridone and weevil-treated plants and lowest for 2,4-D treated plants. Above ground dry biomass was highest for control and weevil-treated plants and lowest for 2,4-D and fluridone-treated plants; the root dry mass of control plants was greater than all treated plants on the final day of the experiment (*P* < 0.01). Lastly, both the number of apical meristems and the average stem length were significantly greater in control plants than the treated plants at the end of the experiment (*P* < 0.01). No significant differences were found in root length between the control and weevil or fluridone treatments; roots were absent in 2,4-D treated plants.

**Table 1:** *F*-values of two-way ANOVAs with treatment (trt), (trt), date of sample collection (DAT), treatment by DAT interaction (trt × DAT).

		Shoot				Roots		
		trt	DAT	trt by DAT		trt	DAT	trt by DAT
				
		df = 3	df = 3	df = 6		df = 3	df = 3	df = 6
Log (wet mass)		5.67^∗^	2.83	17.26^∗∗∗^		5.68^∗^	1.91	11.93^∗∗∗^
Log (dry mass)		4.40^∗^	10.47^∗∗∗^	15.79^∗∗∗^		2.91	5.18^∗∗^	12.13^∗∗∗^
Apical meristems		20.73^∗∗∗^	18.15^∗∗∗^	12.81^∗∗∗^				
Maximum length		5.34^∗^	2.59	8.50^∗∗∗^		2.18	2.42	1.44

*Degrees of freedom are: treatment (df = 3), date (df = 3), trt × date (df = 6), dferror = 24–27, depending on various missing samples. Significance at ^∗^P < 0.05, ^∗∗^P < 0.01, ^∗∗∗^P < 0.001. df_error_ = 27, except for dry mass and apical meristems, where df_error_ = 26.*

### Plant Chemistry

**FIGURE 3:**
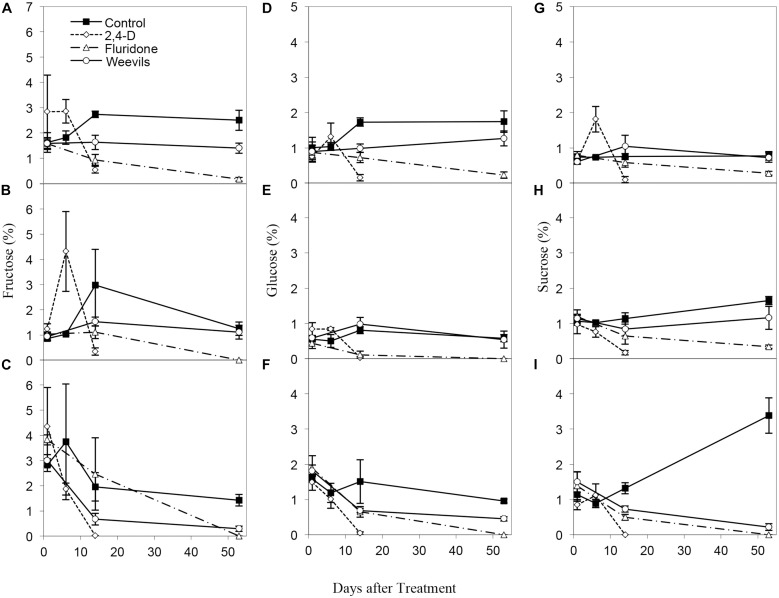
Carbohydrate content of **(A)** tips (34.60 ± 3.72 mg/g dry wt.; mean ± 1 SE), **(B)** middle stems (41.30 ± 4.03 mg/g), and **(C)** roots (38.93 ± 8.71 mg/g) of *M. spicatum* in control and treated plants. Starch content of **(D)** tips (109.59 ± 14.02 mg/g), **(E)** middle stems (117.17 ± 14.12 mg/g), and **(F)** roots (48.11 ± 5.9 mg/g) of *M. spicatum* in control and treated plants. Polyphenol content of **(G)** tips (25.68 ± 4.60 mg/g), **(H)** middle stems (23.36 ± 3.40 mg/g), and **(I)** roots (11.86 ± 1.43 mg/g) of *M. spicatum* in control and treated plants.

Percent carbohydrates and starch differed significantly by treatment for all analyzed plant parts (Table 2). The ANOVAs of plant chemistry parameters of milfoil roots and shoots (tips and middle stem) from the control and treatment tanks include polyphenols, ash, and carbon content were statistically equivalent among the different treatments (Table 2). Carbohydrates were greatest in control-treated tips, stems and roots, moderate in weevil-treated tips and stems and least in fluridone-treated tips and stems and fluridone and weevil-treated roots (Figure 3). The sugars, fructose, glucose and sucrose followed a similar pattern (Supplementary Figure S1 and Supplementary Table S1). Starch was greatest in weevil-treated tips, control-treated stems and roots, moderate in control-treated tips, and weevil-treated stems and roots and least in fluridone-treated tips, stems and roots (Figure 3). Differences observed in percent carbon reflect the differences observed in carbohydrates and starch. In addition, percent nitrogen increased somewhat for middle stems during the mid-point of the exposure period but then decreased by the end of the trial (data not shown). Percent ash varied little throughout the experiment. Polyphenol content tended to decrease in the stem tips and roots during the experiment but no significant differences existed among the treatments (Figure 3).

**Table 2:** *F*-values of the two-way ANOVAs with treatment, DAT and their interaction as factors for *M. spicatum* chemical parameters.

		Tips				Middle				Roots		
		trt	DAT	trt × DAT		trt	DAT	trt × DAT		trt	DAT	trt × DAT
C		10.89^∗∗^	1.72	3.78^∗∗^		1.28	0.85	1.45		1.87	1.65	0.47
N		2.65	2.09	1.04		1.41	10.27^∗∗∗^	0.72		1.07	2.00	2.00
C:N		5.85^∗^	7.34^∗∗^	2.10		1.57	14.55^∗∗∗^	1.05		0.97	0.72	1.03
Ash and Organics		1.07	5.47^∗^	0.43		1.10	8.61^∗∗^	3.20^∗^		Sample size too small		
Carbs		3.87^∗^	2.67	8.96^∗∗∗^		18.52^∗∗∗^	2.11	31.58^∗∗∗^		9.31^∗∗^	12.83^∗∗∗^	2.52^∗^
Starch		9.65^∗∗^	15.71^∗∗∗^	5.82^∗∗∗^		23.32^∗∗∗^	24.27^∗∗∗^	5.73^∗∗∗^		4.21^∗^	20.98^∗∗∗^	6.32^∗∗∗^
Phenols		1.98	8.18^∗∗∗^	2.88^∗^		1.29	2.48	0.77		2.98	2.02	0.64

*C, percent carbon; N, percent nitrogen; C:N, carbon:nitrogen ratio; Carbs, percent carbohydrates; starch, percent starch; Ash, percent ash; organics, percent organics; phenols, percent polyphenols; trt, treatment; DAT, date of sample collection; treatment by DAT interaction (trt × DAT). Degrees of freedom are: treatment (df = 3), date (df = 3), trt × date (df = 6), df_error_ = 22–27, depending on various missing samples, except for Ash, where sample size was very low and df_error_ = 10 (tips) and 16 (middle). Significance at ^∗^P < 0.05, ^∗∗^P < 0.01, ^∗∗∗^P < 0.001.*

## Discussion

The 2,4-D, fluridone, and weevil treatments reduced the growth, carbohydrate and starch content of *M. spicatum*. These findings agree with data from the literature regarding the time course of control achieved with each of the herbicide treatments. The systemic herbicide 2,4-D is listed for control on broadleaf or eudicot plants in both terrestrial and aquatic systems. It acts as a plant growth regulator; by mimicking the activity of normal auxin, 2,4-D causes excessive respiration and cell growth, followed by rapid depletion of carbohydrate reserves and plant death (Green and Westerdahl, 1990; Parsons et al., 2001; Bugbee et al., 2003). This was observed for our experiment, where 2,4-D treatments caused an immediate burst of fructose and sucrose concentrations in plant tips, then a reduction of carbohydrate content in the roots after 5 days and rapid losses of carbohydrates in above ground tissue by 14 days. Both above and below ground starch content were reduced in 2,4-D treated plants by 5 days.

Fluridone is also a systemic herbicide, although the mode of toxicity and time course of control is quite different from 2,4-D. Fluridone disrupts the biosynthetic pathway for the chlorophyll protecting carotenoids, effectively shutting down photosynthesis (Böger, 1996). New plant tissue is often pale or bleached; the ultimate cause of plant death is essentially starvation (Sprecher et al., 1998). Although the loss of photosynthetic potential can be detected shortly after fluridone exposure (Sprecher et al., 1998), actual plant death can take as long as 1–2 months. Fluridone-treated *M. spicatum* experienced a gradual loss of carbohydrate content over the course of the experiment, but plant death did not occur until 50 days after treatment.

Weevils also reduced the growth and carbohydrate composition of *M. spicatum*. Newman et al. (1996) found that weevil stocking reduced total non-structural carbohydrate and sugar content. By day 55 of this experiment, carbohydrate content of control tips, stems and roots was greater than that of weevil-treated plants. When broken down by sugar type, weevil-treated plants had lower fructose and glucose content in plant tips and roots, but similar content in plant stems. Sucrose, often used to transport carbohydrates, was equivalent in control and weevil-treated plant tips and stems, but much lower in weevil-treated roots. One possible explanation is that sugar allocation was impaired by the weevil’s stem mining; sugars were produced, but not translocated to growing tips or roots. Unlike glucose and fructose, the starch content of weevil-treated meristems tips was greater than that of control-tips. This puzzling result could be in preparation of the production autofragments (Madsen, 1997). Because the sugars could not be translocated to roots, plants actively produce autofragments instead, potentially leading to the spread of *M. spicatum* and the production of new plants. In some meristems, the initiation of roots was observed.

The movement of carbohydrates (sugars and starch) highlighted the similarities and differences in resource allocation between herbicide and stem-mining treatments. The carbohydrate content in roots was equivalent for weevil-treated and fluridone-treated plants, which were both low by late summer. Madsen (1997) suggested herbicides should be applied when carbohydrates are at their minimum concentration, often occurring just before regrowth in the spring. However, he noted that for some populations, a late season minimum might also be found at which point herbicides may also be maximally effective (Madsen, 1997). Late summer minima in carbohydrate content has been observed in some milfoil populations from Minnesota lakes (Newman and Biesboer, 2000) and in other plant species (Madsen and Owens, 1998; Marko et al., 2015). This study showed that weevils and herbicides applied during early summer growth could limit carbohydrate allocation to roots in the fall, which should reduce *M. spicatum* growth the following Spring.

A notable difference in weevil and herbicide treatments was the higher carbohydrate content in weevil-treated plant tips and stems. This indicated that while fluridone treated plants stopped producing sugars and slowly used up their reserves, weevil-treated plants were left with starch in the plant tips, which could be used to make fragments. Our treatments indicate that weevil biocontrol may increase autofragmentation of *M. spicatum*, thereby leading to its spread; further impacting native plant communities. While both fluridone and weevils may harm future plant growth, only herbicides prevented autofragmentation.

Controlled laboratory studies suggest that 1–2 mg/L of 2,4-D for a 24–48 h period will result in complete *M. spicatum* control (Getsinger and Netherland, 1997). Under field conditions, salt and butoxyethylester formulations of 2,4-D have been used to effectively manage *M. spicatum* (Bugbee and White, 2005), as well as other problematic plants such as variable milfoil (*M. heterophyllum* Michx.) (Haloragaceae) (Bugbee et al., 2003) and water chestnut (*Trapa natans* L.) (Trapaceae) (Poovey and Getsinger, 2007). Although whole-lake treatments are typically recommended, such efforts can be cost-prohibitive. It is noteworthy that several of the field studies listed above involved spot applications in larger lakes. As a result, measured aqueous concentrations of 2,4-D are often well below the laboratory obtained control values of 1–2 mg/L; in fact, concentrations as low as 0.10 mg/L have still been associated with significant weed death (Bugbee and White, 2005). In our microcosm trials, the highest measured aqueous concentration of 2,4-D was 0.55 mg/L and by day 20 the concentration had dropped to approximately 0.30 mg/L. Nonetheless, nearly complete control of *M. spicatum* was observed in these microcosms, suggesting that concentrations of less than 1.0 mg/L are likely to be effective. Use of low herbicide concentrations may suggests that spot treatments and lower whole-lake herbicide concentrations may be effective management tools in promoting a healthy aquatic plant community and help to alleviate concerns regarding herbicide use.

Reductions in photosynthetic potential can be detected within a few days of fluridone exposures as low as 0.002–0.005 mg/L (Sprecher et al., 1998). However, the product label suggests aqueous concentrations of 0.010–0.070 mg/L for at least 2 months. Netherland et al. (1997) noted that in a mesocosm study, fluridone at 0.005 mg/L could exert greater than 90% control on *M. spicatum* over 90 days without significantly impacting several non-target species but that at concentrations of 0.010–0.020 mg/L, impact on other species became significant. Bugbee and White (2005) noted that in a field experiment with spot applications of fluridone, cabomba (*Cabomba caroliniana* A. Gray) (Cabombaceae) could be effectively controlled at concentrations as low as 0.006 mg/L, but only if those levels were sustained for 60–70 days by repeated or “bump-up” applications. Interestingly, this level of fluridone exposure had no impact on the low growing robbins pondweed (*Potamogeton robbinsii* Oakes) (Potamogetonaceae) within the treatment area but did significantly reduce non-target lily pads that were also present. In the current microcosm trials, concentrations peaked within the first 2 days and began to decrease quickly; as in the field study of Bugbee and White (2005), a “bump-up” application was necessary to maintain suitable levels. In agreement with the literature, an average concentration of approximately 0.025 mg/L resulted in 75% control over the course of the 60 day trial.

The greater values for biomass, stem length and apical meristem numbers found for control plants clearly indicate that the weevil- and herbicide-treated plants were harmed by the treatments. The milfoil treated with 2,4-D were most negatively affected. Weevil- and fluridone-treated plants were very similar in terms of their measured physical responses but statistically significant differences were observed in their morphological and chemical properties. In particular, fluridone-treated plants lost photosynthetic pigments and capability, followed by stunted growth. Conversely, weevil-treated plants maintained photosynthetic potential but affected meristems were consumed and stems were hollowed out. This could result in weevil-treated plants producing autofragments, whereas fluridone-treated plants did not. Depending on the spread of the invasive species within the lake, herbicide treatments may be; more effective at reducing the potential for the spread of *M. spicatum*. However, herbicide treatments often must be applied annually for several years before the invasive plant is brought to acceptable levels; whereas biological controls may only need to be applied once and are species specific (Hussner et al., 2017).

The use of the milfoil weevil for the biological control of *M. spicatum* is considered augmentative biocontrol because a native herbivore is being supplemented (Hussner et al., 2017). While the control of emergent and floating-leaf aquatic plants has been successful, the control of submersed aquatic plants has been more difficult (McFadyen, 1998; Newman, 2004; Hussner et al., 2017). As seen in this study, biocontrol organisms can provide localized biomass reduction. Unfortunately, those short-term impacts do not always translate to long-term control (Newman, 2004). Biocontrol populations can fluctuate year to year due to environmental factors such as poor overwintering habitat, fish predation and difficulty with migration to healthy weed patches (Newman, 2004; Hussner et al., 2017). Hussner et al. (2017) suggests that biological control organisms for submersed aquatic plants are best considered as part of an integrated pest management plan.

The differences observed among the treatments in plant carbohydrate and starch content highlight the different impacts that herbicides and weevils have on milfoil plants. While 2,4-D-treated plants simply die off and the concentrations of both starch and carbohydrates decline dramatically, the differences between the fluridone-and weevil-treated plants are more subtle. Carbohydrate content in weevil-treated tip and middle stems remains higher than fluridone-treated plants throughout the experiment, but carbohydrate content in roots in weevil- and fluridone-treated plants is similarly quite low. This demonstrates the different modes of action in fluridone and weevil treatments. Fluridone directly impacts photosynthetic potential, which obviously results in carbohydrate reduction over time. Conversely, weevils do not impact carbohydrate production, but do impact its translocation. Both effects result in lower starch content in the roots, which would subsequently result in lower over-winter survival.

In addition to production and translocation of carbohydrates, the production of defensive chemicals can indicate how well-defended the plant is against herbivores, and therefore how susceptible it is to biological control organisms. Polyphenol production is a common defensive mechanism of plants and these chemicals are known to be abundant in milfoils (Marko et al., 2008; Fornoff and Gross, 2014). Polyphenol concentrations varied only slightly over time in *M. spicatum* tips and were not different by treatment for any plant part suggesting polyphenol production is constitutive or not induced by herbivory of the specialist weevil. As *E. lecontei* is a milfoil specialist, it is unlikely to be impacted by polyphenol content (Marko and Newman, 2017). However, polyphenols due play an important role in the defense of *M. spicatum* against algae (Gross et al., 1996) and the generalist *A. ephemerella* (Fornoff and Gross, 2014).

Based on our results, the milfoil weevil would be a more effective control when used with fluridone than with 2,4-D. The herbicide 2,4-D resulted in the rapid die-back of *M. spicatum*, whereas plants treated with fluridone were healthier longer. Fluridone has been used as an effective control of invasive submersed plants while maintaining healthy native plant communities (Bremigan et al., 2005; Pedlow et al., 2006). However, fluridone treatments have also resulted in the decline of native plant communities (Valley et al., 2006; Parsons et al., 2009) or resulted in increased invasive plant growth several years after treatment (Wagner et al., 2007). The use of *E. lecontei* with fluridone could result in the biomass reduction of *M. spicatum.* The longer senescence times when using fluridone may provide the milfoil weevil with enough opportunity to find healthy milfoil. Spot-treatments of fluridone leaving nearby healthy *M. spicatum* populations would provide the best chance for successful integration of herbicide and biocontrol organisms as the slow senescence of fluridone and the nearby healthy population of *M. spicatum* would provide *E. lecontei* with the time to move to a refuge.

## Conclusion

In conclusion, *M. spicatum* was effectively controlled by both chemical and biological methods. The herbicide 2,4-D resulted in rapid and complete weed death, whereas the herbicide fluridone and the biological control agent *E. lecontei* yielded 70–75% control during the 53 day trial. Although total biomass remaining was similar between fluridone- and *E. lecontei*-treated plants, clear biochemical differences among the surviving vegetation could be detected and these differences were directly related to differential modes of action. While all treatments did reduce *M. spicatum* biomass, no one treatment stood out as the most effective way to control *M. spicatum* for the promotion of native species growth. Both herbicides show mixed results against native vegetation in field trials and both often result in long-term regrowth of the invasive species. *E. lecontei* alone does not seem to adequately control *M. spicatum* on a yearly basis, but it does provide the only possibility of long-term control and has no negative non-target impacts. The most effective long-term control to of *M. spicatum* to promote the growth of native vegetation is likely an integration of management choices.

## Author Contributions

All authors equally participated the all aspects of this project from design, data collection, and analysis to drafting of this manuscript.

## Conflict of Interest Statement

The authors declare that the research was conducted in the absence of any commercial or financial relationships that could be construed as a potential conflict of interest.

## Supplementary Material

FIGURE S1 Fructose content of **(A)** tips (1.70 ± 0.24 mg/g dry wt.; mean ± 1 SE), **(B)** middle stems (1.36 ± 0.31 mg/g), and **(C)** roots (2.03 ± 0.41 mg/g) of *M. spicatum* in control and treated plants. Glucose content of **(D)** tips (0.98 ± 0.13 mg/g), **(E)** middle stems (0.52 ± 0.088 mg/g) and **(F)** roots (1.02 ± 0.17 mg/g) of *M. spicatum* in control and treated plants. Sucrose content of G) tips (0.74 ± 0.11 mg/g), **(H)** middle stems (0.94 ± 0.11 mg/g) and **(I)** roots (1.00 ± 0.24 mg/g) of *M. spicatum* in control and treated plants.

TABLE S1 *F*-values of the two-way ANOVAs with treatment, DAT and their interaction as factors for *M. spicatum* chemical parameters.
