# Supplementary figures and images for: Direct Comparison of Herbicidal or Biological Treatment on Myriophyllum spicatum Control and Biochemistry
Source: Front Plant Sci. 2018 Dec 10;9:1814. doi: 10.3389/fpls.2018.01814 (PMC6295576; doi:10.3389/fpls.2018.01814)

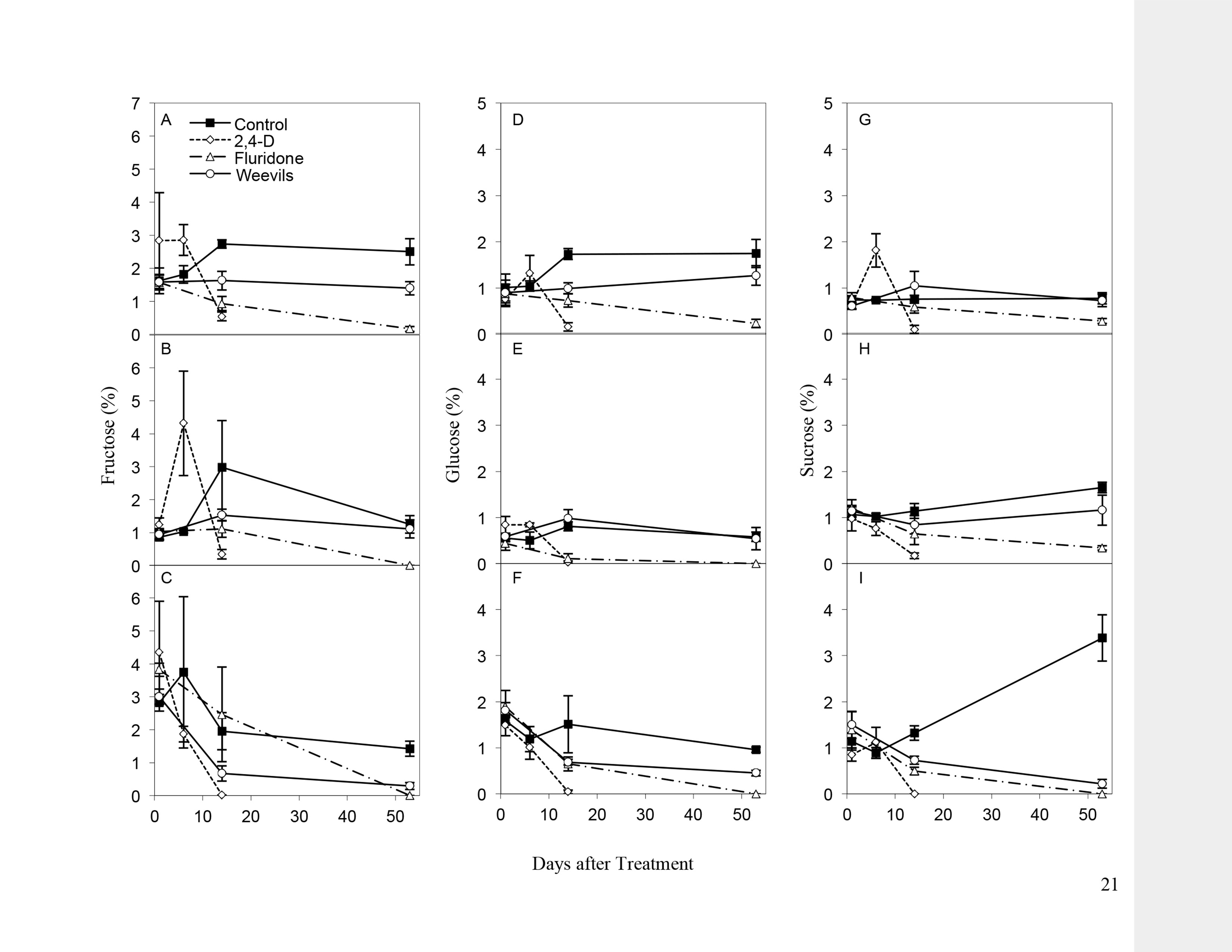

Supplement: FIGURE S1 — Fructose content of (A) tips (1.70 ± 0.24 mg/g dry wt.; mean ± 1 SE), (B) middle stems (1.36 ± 0.31 mg/g), and (C) roots (2.03 ± 0.41 mg/g) of M. spicatum in control and treated plants. Glucose content of (D) tips (0.98 ± 0.13 mg/g), (E) middle stems (0.52 ± 0.088 mg/g) and (F) roots (1.02 ± 0.17 mg/g) of M. spicatum in control and treated plants. Sucrose content of G) tips (0.74 ± 0.11 mg/g), (H) middle stems (0.94 ± 0.11 mg/g) and (I) roots (1.00 ± 0.24 mg/g) of M. spicatum in control and treated plants. [file Image_1.jpg]
