# Supplementary material for: Direct Comparison of Herbicidal or Biological Treatment on Myriophyllum spicatum Control and Biochemistry
Source: Front Plant Sci. 2018 Dec 10;9:1814. doi: 10.3389/fpls.2018.01814 (PMC6295576; doi:10.3389/fpls.2018.01814)
Supplement: TABLE S1 — F-values of the two-way ANOVAs with treatment, DAT and their interaction as factors for M. spicatum chemical parameters. [file Table_1.docx]

Supplementary Table 1. F-values of the two-way ANOVAs with treatment, DAT and their interaction as factors for *M. spicatum* sugar percentages.

|  |  | Tips | | |  | Middle | | |  | Roots | | |
| --- | --- | --- | --- | --- | --- | --- | --- | --- | --- | --- | --- | --- |
|  |  | trt | DAT | trt × DAT |  | trt | DAT | trt × DAT |  | trt | DAT | trt × DAT |
|  |  |  |  |  |  |  |  |  |  |  |  |  |
| Fructose |  | 3.74 | 0.87 | 3.35^*^ |  | 0.70 | 2.78 | 4.14^**^ |  | 2.46 | 9.93^***^ | 1.31 |
| Glucose |  | 10.20^**^ | 0.46 | 5.01^**^ |  | 4.48^*^ | 3.04^*^ | 8.54^***^ |  | 3.59 | 24.46^***^ | 1.64 |
| Sucrose |  | 0.95 | 9.84^***^ | 8.45^***^ |  | 6.53^*^ | 3.43^*^ | 3.79^**^ |  | 27.84^***^ | 4.57^*^ | 15.26^***^ |

Treatment (trt), date of sample collection (DAT), treatment by DAT interaction (trt × DAT). Degrees of freedom are: treatment (df=3), date (df=3), trt × date (df=6), df_error_=24-27, depending on various missing samples. Significance at ^*^P<0.05, ^**^P<0.01, ^***^P<0.001
